# Maternal IgA2 Recognizes Similar Fractions of Colostrum and Fecal Neonatal Microbiota

**DOI:** 10.3389/fimmu.2021.712130

**Published:** 2021-11-04

**Authors:** Erick Sánchez-Salguero, Karina Corona-Cervantes, Hector Armando Guzmán-Aquino, María Fernanda de la Borbolla-Cruz, Víctor Contreras-Vargas, Alberto Piña-Escobedo, Jaime García-Mena, Leopoldo Santos-Argumedo

**Affiliations:** ^1^ Department of Molecular Biomedicine, Center for Research and Advanced Studies of the National Polytechnic Institute (CINVESTAV-IPN), Mexico City, Mexico; ^2^ Department of Genetics and Molecular Biology, Center for Research and Advanced Studies of the National Polytechnic Institute (CINVESTAV-IPN), México City, Mexico; ^3^ Department of Gynecology Regional Hospital “October 1^st^”, Institute for Security and Social Services of State Workers (ISSSTE), México City, Mexico

**Keywords:** IgA2, IgA1, microbiota, maternal transfer, IgA-seq, colostrum

## Abstract

Microbiota acquired during labor and through the first days of life contributes to the newborn’s immune maturation and development. Mother provides probiotics and prebiotics factors through colostrum and maternal milk to shape the first neonatal microbiota. Previous works have reported that immunoglobulin A (IgA) secreted in colostrum is coating a fraction of maternal microbiota. Thus, to better characterize this IgA-microbiota association, we used flow cytometry coupled with 16S rRNA gene sequencing (IgA-Seq) in human colostrum and neonatal feces. We identified IgA bound bacteria (IgA+) and characterized their diversity and composition shared in colostrum fractions and neonatal fecal bacteria. We found that IgA2 is mainly associated with *Bifidobacterium*, *Pseudomonas, Lactobacillus*, and *Paracoccus*, among other genera shared in colostrum and neonatal fecal samples. We found that metabolic pathways related to epithelial adhesion and carbohydrate consumption are enriched within the IgA2+ fecal microbiota. The association of IgA2 with specific bacteria could be explained because these antibodies recognize common antigens expressed on the surface of these bacterial genera. Our data suggest a preferential targeting of commensal bacteria by IgA2, revealing a possible function of maternal IgA2 in the shaping of the fecal microbial composition in the neonate during the first days of life.

## Introduction

Neonatal acquisition of an adequate microbiota composition during early life (1) is regulated by various conditions like birth route (2), lactation mode (3), mother’s diet, and maternal intestinal microbiota (4). Colostrum, the first secretion during lactation, contains factors contributing to the newborn’s defense, immune stimulation, and it is an essential source of microorganisms for the first microbiota in the neonate (5, 6). These microorganisms live along the breast duct (7), increasing their composition and variability near the nipple and decrease near to mammary acinus (8, 9).

Surprisingly, from an epidemiological point of view, some of these colostrum bacterial genera, have potential pathogenic properties; being a stimulus and signal for the development and maturation of the newborn’s intestinal innate immunity (10, 11). Early interaction with microorganisms appears to play an essential role in developing the infant immune system, establishing a tolerance response to these bacterial species (12–14).

During the first moments of life, the human colostrum is the primary source of maternal IgA. Maternal IgA recognizes and associates with resident microbiota in acini, duct, and nipple skin during colostrum ejection to transit through the newborn gastrointestinal tract, and it can be recovered in the newborn’s feces (15–17).

In the intestine, IgA neutralizes pathogenic microorganisms or may directly regulate microbiota composition (18–20), suggesting that maternal IgA could impact neonatal intestinal microbiota composition with different mechanisms in the neonatal intestine (21, 22). These paradoxical functions of the IgA could be explained with IgA origin: IgA produced by T-dependent response recognizes potential pathogens. Meanwhile, IgA produced by the T-independent mechanism mainly recognizes commensal microbiota species (23, 24).

Human IgA has two subclasses, IgA1 and IgA2, with well-defined properties and structures (25). Sterlin and colleagues described that most commensal bacteria present in adult fecal samples are associated with IgA2, suggesting that this subclass would play a role in regulating commensal bacteria under homeostatic conditions (26). Different studies have described an individual variability in specificity between colostrum IgA subclasses, where the IgA2 subclass mainly recognizes glycans on the surface of bacteria in the intestine tract. IgA2 subclass production is mainly induced by a T-independent mechanism (27, 28).

These IgA could shape microbiota composition in the intestine by direct association with specific bacteria genera and indirectly to the generation of niches and interactions between IgA+ bacteria, and other microbial genera (16, 17) essential to begin the formation of a new microbiota for the neonates. However, despite that maternal IgA could shape the first microbiota in the intestine of the neonate, the distribution and composition of IgA1 *vs.* IgA2 -coated bacteria in colostrum and their role in transferring maternal microbiota to the newborn has not been described.

In this work, we analyzed colostrum from mothers and fecal samples from neonates (before and after feeding with colostrum) to compare microbiota composition. Our results demonstrated a preferential targeting of commensal bacteria by IgA2 during the first days of life. IgA2 in the colostrum could play a role and to influence in the bacterial composition in the neonate´s fecal microbiota community.

## Materials and Methods

### Design and Type of Study

This study is analytical, observational, prospective, and longitudinal. The population was selected through a simple random process. Clinically healthy women were recruited at Hospital Regional 1° de Octubre (HR 1° Oct) Instituto de Seguridad y Servicios Sociales de los Trabajadores del Estado (ISSSTE). All the activities detailed in this protocol have the approval of the Research and Bioethics Committee of HR 1° Oct (090201/14.1/086/2017). Sociodemographic, anthropometric, and clinical data (education, occupation, maternal and gestational age, and type of delivery) were recorded. Two to three milliliters of colostrum and 0.5-2 grams of fecal samples were collected from each mother or newborn child, respectively. One hundred eighty samples from ninety-nine mother-newborn binomials were collected. The sample size was determined by a non-probabilistic convenience sampling, considering new delivery mothers between November 2017 and October 2019.

### Selection Criteria

Inclusion criteria. Women between 25 and 30 years old, with a full-term pregnancy (38-42 weeks of gestation), due to an exclusive vaginal delivery, who regularly attended their medical care to monitor their pregnancy, deliver the unique product, and agree to participate this project.

Exclusion criteria. Women who had C-section delivery, or were unable or unwilling to breastfeed, who reported chronic degenerative disease under drug, hormonal, or antibiotic treatment were omitted. Women were also omitted if they reported alcohol and smoking during pregnancy, decided not to continue in the study, or declared an acute illness in the last three weeks before delivery.

### Sampling

Before delivery, patients who agreed to participate in the study received informed consent, which they read and signed following the Helsinki treaty, General Health Law, and Hospital Ethics Committee. They were given a questionnaire, which they filled out with their general data and clinical information. Data reported were corroborated with their medical history files from the hospital archive. Since delivery, babies were monitored with care to detect the first fecal production (meconium). Before breastfeeding, twenty representative meconium samples were obtained. At that moment, nappies were removed and placed in sterile plastic transport bags. External portions of meconium were removed, and the central portion was scraped with a sterile tongue depressor to avoid contamination from external portions with neonate’s skin, nappy, or environment. Samples were then taken from the nappies in a laminar flow cabinet using aseptic techniques. These twenty different meconium samples from different and no related babies were polled in groups of four samples, to be polled individually in five different meconium mixes. These five mixed samples were processed to determine IgA presence and obtained bacteria fractions to be sequenced, as we indicated below. We used mixed samples to secure enough bacterial quantity for subsequent experiments.

Thirty-six colostrum samples were obtained following the procedures described in the milk extraction technique protocol in the lactation clinics of Children’s and General Hospitals, within the first two hours postpartum and before nursing babies. The nipple and areola area were cleaned with physiological saline solution and sterile gauze. Special care was taken to ensure infants were exclusively breastfed during their permanence in the Hospital. After three days, a stool sample from each of the forty-six neonates was collected. Pasteurized maternal milk (Pasteurized) from the Hospital’s Milk Bank was used as a control to compare with milk freshly delivered from the breast. This pasteurized milk is a supplementary feeding option recommended when breastfeeding is not feasible; for example, ablactation, maternal disease, or impediment due to drug intake. This pasteurized sample consists of milk from donor mothers less than seven days postpartum in Hospital Milk Bank, pasteurized (63°C for 30 min), and delivered to the neonates (29). Pasteurization eliminates most bacteria and denatures IgA, then newborn fed with pasteurized maternal milk will neither receive IgA nor IgA-coated microbiota. Moreover, neonates do not produce IgA; thus, all IgA present in their feces must come from the maternal colostrum. After treatment, 2 mL of twenty-eight samples were taken. Finally, sampling feces from a group of twenty-seven neonates fed with pasteurized milk was included according to the pediatrician’s indications. Samples were kept at 4°C for their transfer to the laboratory, to be processed within the first 20 minutes after sampling.

### Phase Separation

For separation, the methodology applied in this work was previously reported by Bunker et al. (23). Briefly, 500 mg meconium/stool samples were taken and resuspended in 2 mL of sterile phosphate buffer saline (PBS) (Cat# P4417-100TAB, Sigma-Aldrich^®^, St Louis, Missouri, USA). Colostrum, meconium, and stool samples were treated with 1% protease inhibitor cocktail (Cat #P8465, Sigma Aldrich^®^) with 0.01% sodium azide (NaN_3_) (Cat# S2002, Sigma-Aldrich^®^) following manufacturer specifications. Samples were centrifuged 10 minutes at 400 g and 4°C, and three phases were obtained: a) a top lipid phase, b) a middle aqueous phase, and c) cellular components in the bottom. The aqueous phase was separated without dragging the surface phase’s remains or the cellular components. These top and bottom phases were discarded. The aqueous phase was centrifugated for 30 minutes at 8,000 g and 4°C (Allegra X-22 Series Beckman Coulter^®^ USA centrifuge). Two phases were obtained, supernatant and pellet. The supernatant was stored in aliquots at -70°C for the determination of free immunoglobulins by quantitative ELISA assay. The pellet was resuspended and filtered through a 0.45-micron filter to separate membrane bodies and exosomes (30). The residue, rich in bacterial cells, was recovered and processed to separate the bacteria coated with IgA subclasses (IgA1+ and IgA2+) or free fraction (IgA-).

### Quantitative ELISA Assay

The method was previously reported by Sánchez-Salguero et al. (31). Briefly, flat-bottom 96-well polystyrene plates (Thermo scientific^®^ MaxiSorp USA) were coated with 100 µL of mouse monoclonal antibody 7A09 anti-human light chain (Cat# ab1942, Abcam^®^, Cambridge UK), at 1 µg/mL in PBS. The plates were incubated 12 hours at 4° C. Blocking was carried out with 200 µL per well bovine serum albumin (BSA) (Cat# A1933, Sigma-Aldrich^®^) 5% in PBS with Tween 20 (Cat# P2287, Sigma-Aldrich^®^) 0.05% (PBST), incubating 90 min at 25°C. For the standard curve, 100 µL of the stock solutions prepared from commercial reagents of purified human immunoglobulins were added per well: human IgA1 protein (Cat# ab91020, Abcam^®^) and human IgA2 protein (Cat# ab91021, Abcam^®^) in PBS; incubating 2 hours at 37°C. For the detection of IgA1, a biotin-coupled anti-human IgA1 Fc region mouse monoclonal antibody was added (Cat# ab99796, Abcam^®^) at 1:2,000 dilution. For detection of IgA2, a biotin-coupled anti-human IgA2 Fc region mouse monoclonal antibody was added (Cat# ab128731, Abcam^®^) at 1:1,000 dilution. Finally, 100 µL per well of a 1:5,000 dilution of streptavidin-horseradish peroxidase (HRP) complex (Cat# ab7403, Abcam^®^) were added and incubated 1 hour at 37°C. For development, 100 µL per well of 3’3’’-5-5-tetramethylbenzidine (TMB) with hydrogen peroxide (H_2_O_2_) (Cat# ab171523, Abcam^®^) was incubated 10 min. The reaction was stopped with the addition of 100 µL of 0.2 M sulfuric acid (H_2_SO_4_) (Cat# 7664-93-9 JT Baker^®^, Fisher Scientific, USA). The plates were read in the spectrophotometer (Sunrise absorbance reader, Tecan’s Magellan^®^ universal reader) at 450 nm. The analysis was performed using GraphPad Prism^®^ version 7.00 (GraphPad^®^ Software, La Jolla, California USA).

### Flow Cytometry Controls

Aliquots from each bacterial pellet isolated were analyzed by flow cytometry to determine whether size and complexity had the characteristics of typical bacteria cells compared with the *E. coli* strain as a reference. According to the supplier’s instructions, we used a Bacterial Viability Assay Kit (Cat# ab189618, Abcam^®^) to avoid false nonbacterial artifacts detection. In brief, for viability assay, 3 µL of a mixture with equal volumes of SYTO^®^ and propidium iodide (PI) was added for each milliliter of the bacterial suspension, incubated 15 minutes in the dark at room temperature. Finally, the mixture was washed three times with PBS and analyzed by a flow cytometer (**Supplementary Figure 1A**).

To discard the presence of exosome particles in the fractions, we stained the samples with a commonly used exosome marker, CD81 (Cat# 349514, Biolegend^®^, San Diego, CA, USA) (32). (**Supplementary Figure 1B**).

In addition, we included biotinylated mouse anti-human IgE as an isotype control of IgA staining and purification. We proceeded with the staining protocol, as described below: Briefly, bacterial samples were blocked with PBS containing 0.25% of BSA, 5% fetal calf serum (FCS) (Cat# 26140, Thermo Fisher^®^), and 2 mM of Ethylenediaminetetraacetic acid (EDTA) (Cat# 6381-92-6, JT Baker^®^, Phillipsburg, NJ, USA), then washed and incubated with a biotinylated mouse anti-human IgE (Cat # ab99807, Abcam^®^). Bacterial preparation was washed and split into two samples for staining with APC Cy7-streptavidin or FITC-streptavidin. After washing, these preparations were analyzed by flow cytometry (**Supplementary Figure 1C**).

To demonstrate the specificity of the staining, polystyrene microbeads (Cat # LB30-1ML, Merck Millipore^®^, EE. UU.) coated with purified human IgA1 (Cat # ab91020, Abcam^®^) or IgA2 (Cat # ab91021, Abcam^®^) were used as positive controls as previously reported (33, 34). Each fraction of microbeads was independently analyzed with biotinylated mouse anti-human IgA1 (Cat # ab99796, Abcam^®^) or anti-IgA2 (Cat # ab128731, Abcam^®^). After staining with streptavidin-APCy7 (Cat# 405208, Biolegend^®^) or streptavidin- FITC (Cat# 405201, Biolegend^®^), the microbeads were washed and then analyzed by flow cytometry (**Supplementary Figure 1D**).

### Separation of Bacteria Into IgA1+ and IgA2+ Fractions

According to their association with IgA subclasses, pellets were processed to separate bacteria fractions, as previously reported (23). The pellets were incubated for fifteen minutes at room temperature with PBS containing 0.25% BSA, 5% FCS, and 2 mM EDTA. Subsequently, they were washed five times with PBS and resuspended in a final volume of 1 mL. Total sample volume was separated into four sterile tubes: a) total bacteria, b) IgA- bacteria, c) IgA1+ bacteria, and d) IgA2+ bacteria. For the separation of the IgA-associated bacterial fractions, the samples were incubated with biotinylated anti-human IgA1 (Cat# ab99796, Abcam^®^) and anti-human IgA2 (Cat# ab128731, Abcam^®^) at a dilution of 1:2500 and 1:2,000, respectively. Subsequently, streptavidin-APCy7 (Cat# 405208, Biolegend^®^) or streptavidin-FITC (Cat# 405201, Biolegend^®^) were added. Finally, magnetic anti-FITC microbeads (Cat# 130-048-701 Miltenyi Biotec Inc., Sunnyvale, CA, USA) or anti Cy7 microbeads (Cat# 130-091-652 Miltenyi Biotec Inc.) were added. Positive selection was carried out using a magnet system for cell separation (Cat# 18000 EasySep™ Magnet, Stem Cell Technologies^®^, Canada). The purity of IgA1+, IgA2+, and IgA- bacterial fractions was verified in the CytoFLEX flow cytometer (Cat# B53000 Beckman Coulter^®^) and analyzed with CytExpert Software (Beckman Coulter^®^) (**Supplementary Figure 2**).

### Enzyme Protease Kinetic Assay

Since IgA subclasses and microbiota biomass coated with each immunoglobulin are in different quantities, we used a similar ratio to determine whether the loss of IgA1 signal was due to bacterial enzymatic degradation activity. We used human IgA1 (Cat# ab91020, Abcam^®^), at 10 mg/mL or human IgA2 (Cat# ab91021, Abcam^®^), at 12 mg/mL diluted in PBS. These proteins were incubated with 100,000 IgA1+ bacteria fraction or 200,000 IgA2+ bacteria fraction isolated from human colostrum; at 4°C (as temperature control) or 37°C. Independently, we included the same fractions with a protease inhibitor cocktail (Cat #P8465, Sigma-Aldrich^®^) (as a negative control). All samples were incubated for up to two hours. At each 20 minutes’ interval, we remove and freeze an aliquot to quantify IgA1 or IgA2 by ELISA, as described above.

### Extraction and Purification of Deoxyribonucleic Acid (DNA)

DNA was obtained from bacteria fractions with Favor Prep Milk Bacterial DNA Extraction Kit (Cat# FASTI 001-1 Favorgen Qiagen^®^, Cambridge, UK) and QIAmp DNA Stool Mini Kit (Cat# 51306 Qiagen^®^); as previously reported (7) and according to manufacturer’s specifications. The concentration and purity of DNA were determined by absorbance ratio 260/280 using Nanodrop Lite Spectrophotometer (Cat# ND-1000 Thermo Scientific^®^), 300 µL of PBS pH 7.4 was used a negative control for DNA extraction. DNA quality was evaluated by electrophoresis in 0.5% agarose gel, using Tris-Borate-EDTA (TBE), at 90 Volts for 50 min. The gels were stained with 0.80 μL of Midori Green dye advanced (Cat# CLS-1909-G Nippon Genetics^®^ Europe, Dueren, Germany) dilution 1:15. The gels were documented by Chemidoc™ XRS+ System Molecular Imager Gel (Cat# 170-8070 Bio-Rad^®^, Hercules CA, USA). DNA was stored at -20°C until library preparation and sequencing protocols.

### Amplification of the V3 Region of 16S rRNA

For each DNA sample, a ~281 base pair (bp) fragment corresponding to the hypervariable region 3 (V3) of the bacterial 16S ribosomal RNA (16S rRNA) was amplified from each DNA sample. Universal primers amplify the V3 polymorphic region of the rDNA gene, V3-341F forward primer (set of barcodes 1-100) complementary to positions 340-356 of the *E. coli* 16S rRNA molecule *rrnB* GenBank https://www.ncbi.nlm.nih.gov/nuccore/J01859 and V3-518R reverse primer complementary to positions 517-533 of same molecule were used, containing the adapter and barcode sequences for multiple samples massive sequencing (6). Amplification was carried out by end-point polymerase chain reaction (PCR) in Applied Biosystems^®^ 2720 Thermal Cycler (ThermoFisher Scientific^®^), working with a final DNA quantity of 50 ng per reaction. Bands of interest were analyzed with the documentation system Molecular Imager^®^ for an expecting fragment size of ~281 bp. DNA amplicon were not observed in all negative controls. PCR conditions were the same as in previous reports (6). However, we included them in the library mix for subsequent massive sequencing, as we clarified below.

### Low Biomass Controls

Since human milk and meconium have low microbial biomass, contamination with laboratory reagents (including nucleic acid extraction kits and PCR master mix solution) was a significant concern with these samples. For this reason, the sampling process was carried out by qualified medical personnel using sterile material. We extreme precautions corroborating that neither the materials nor the procedure contaminated the samples by including several controls. The controls included: “Sampling material” (swabs, tubes, and tongue depressors analyzed under the same conditions as meconium samples). “Environment” (tubes opened in the hospital environment during sampling process), and “Master Mix decontamination”. In all controls, we evaluated the presence of bacteria by Gram staining, then registered in 1000 X Eclipse E4000 optical microscope (Nikon^®^, Japan) by Image-Pro Plus 7.0 (Media Cybernetics^®^, EE. UU.) (**Supplementary Figure 3A**). Bacterial particles were also searched by flow cytometry as described above (**Supplementary Figure 3B**). Finally, all control samples were processed to purify DNA and then to amplify 16S rDNA by PCR (**Supplementary Figure 3C**), as indicated below. For the Master Mix decontamination protocol, we used a double-stranded DNase (ds DNase) to degrade any contaminating DNA that might be present in the PCR mix before adding DNA extracted from the samples (Cat# EN7011, Thermo Fisher^®^) as described previously (35, 36). In brief, PCR master mix solutions (including primers) were treated with 0.5 uL of ds DNase and 0.5 uL of dithiothreitol per 50 uL reaction, then incubated at 37° C for 20 min, followed by 20 min incubation at 60°C, which it was corroborated during the standardization of this assay exposed known quantities of DNA in previously decontaminated PCR mix. Then, decontaminated master mix solutions were cooled on ice for two min to prevent heat-related changes in volume. 16S rRNA amplifications were carried out as previously reported (6). No amplicon was detected in negative controls as corroborated by agarose gel electrophoresis (**Supplementary Figure 3C**) (37). Despite no amplicon detection, we included these controls in a mixed DNA library and were sequenced along with our samples to determine reagent and laboratory contamination impact in the massive sequencing process (38, 39); but Ion Torrent software no reported result. These sequences were barcoded with forwarding primers numbered like F003, F010, F018, F020, F040, F050, and F070.

### High-Throughput DNA Sequencing

Samples with specific barcodes were mixed, and equal mass amounts of each 1-100 barcoded amplicons quantified by gel densitometry were pooled. Amplicons size in the library was verified using the Bioanalyzer equipment. For template preparation, emulsion PCR spheres with Ion Sphere™ Quality Control Kit (Cat# 4468656 Life Technologies^®^, Thermo Fisher Scientific^®^) were conditioned, and massive PCR reactions were implemented in the Thermocycler system. Likewise, the spheres’ quality was verified with the sample sequenced using Ion OneTouch™ system equipment (Cat# 4474779 Thermo Fisher Scientific^®^) and Qubit 2.0 fluorometer Massive sequencing (Cat# Q33327 Thermo Fisher Scientific^®^). Finally, samples’ sequencing was performed by Ion Torrent PGM™ Sequencer equipment (Cat# A25511 Thermo Fisher Scientific^®^), using Ion 318 Chip Kit v2 (Life Technologies^®^, Thermo Fisher Scientific^®^), as previously described (6).

### Analysis of Sequencing Data

Data were processed using Torrent Suite v4.4.3 software (Thermo Fisher Scientific^®^) to exclude polyclonal and low-quality sequences. The reads were assigned to the source sample employing the DNA primers system. The quality control of sequences was performed with FastQC (https://www.bioinformatics.babraham.ac.uk/projects/fastqc/), and all readings were trimmed to 200 nt in length with Trimmomatic v0.36 (http://www.usadellab.org/cms/index.php?page=trimmomatic) to remove low qualities bases from the sequences in according with Ion Torrent equipment report that was up 200 nt. Readings that passed the quality tests were exported as FASTQ files that were later transformed into FASTA files and were analyzed using the QIIME pipeline software version 1.9.1 (40, 41). (http://qiime.org/). DNA sequences were classified into Operational Taxonomic Units (OTUs) using closed-based picking parameters with a 97% similarity level against Greengenes database v13.8 (https://greengenes.lbl.gov/). The sequence and corresponding mapping files for all samples used in this study were deposited in the NCBI BioSample repository PRJNA707069 ID 707069 - BioProject - NCBI (nih.gov). Microbial diversity was calculated using alpha diversity (within samples) and beta diversity (among samples). Alpha diversity was estimated using different indices: observed OTUs, Chao’s index, Shannon’s index, and Simpson’s index. These indices were determined using phyloseq and plotted with ggplot2 packages in the R environment (v3.4.4) (https://r-project.org/). In addition to the controls previously mentioned, we used the data filtration decontam package in R (Version 1.10.0) (https://www.bioconductor.org/packages/release/bioc/html/decontam.html) to avoid inaccurate results of relative bacterial frequencies and to monitor the background contamination. Based on this analysis, we identified and filtered out twenty-nine contaminants bacterial OTUs from the database samples (**Supplementary Figure 4**) as previously reported (42). Despite the considerable number of contaminants OTUs (twenty-nine), These OTUs included bacteria genera mainly from the orders *Clostridiales* and *Pseudomonas* that appeared in an insufficient number of samples, including fecal and low microbial biomass samples. This very low biomass could be the reason for neither amplification of PCR products nor sequencing results obtained for these controls, besides, these null we included in the library mix for massive sequencing. For beta diversity, dissimilarity was estimated using UniFrac analysis (weighted and unweighted). A two-dimensional scatter plot was generated using principal coordinate analysis (PCoA). ANOSIM and Adonis statistical tests were applied. The relative abundance of the bacterial groups identified in the samples was compared by the Wilcoxon rank test using the SPSS software (version 14.0) STAMP - Bioinformatics Software (dal.ca). Data analysis (group comparison) was performed by Student’s t-test or by Mann-Whitney U test. Values of p <0.05 were considered significant. Statistical operations were performed using the SPSS program (version 14.0). Linear discriminate t analysis (LDA) effect side algorithm (LEfSe v1.0) was used to identify statistically significant taxa in different sample groups. We used LDA Fisher’s linear discriminant to estimate each taxon’s effect size between groups. For LDA analysis of bacteria in maternal milk samples and IgA2-associated bacteria in neonate feces fed with colostrum, thresholds 2.0 and 3.5 of significance were used, respectively. PICRUST v1.1.1 (Phylogenetic Investigation of Communities by Reconstruction of Unobserved States) (http://picrust.github.io/picrust/) was used to predict functional metagenomes based on 16S rRNA gene data with the Kyoto Encyclopedia of Genes and Genomes (KEGG) orthologue classification database in hierarchy level 3 pathways. Statistical analysis of Taxonomic and Function software (STAMP v2.1.3) was used to determine significant differences in OTU’s abundance and metabolic pathways. We performed a Source Tracker analysis to predict the origin of OTU’s in each neonatal stool sample using corresponding human colostrum as a potential source to estimate the proportion of bacteria present in fecal samples attributable to the human milk. This analysis was made in the QIIME platform using Source Tracker (v0.9.5) software (43).

### Carbohydrate’s Microarrays

The IgA1 + or IgA2 + bacterial fractions obtained from the colostrum samples were treated with RIPA buffer (Cat # R0278 Sigma) for 30 min at 4°C. Then, the lysate was centrifuged 15 min at 12,000 xg. The supernatant was discarded, and elution buffer (0.1 M Glycine-HCl at pH 2.5-3.0) was added to the pellet to separate the immunoglobulins bound to the remains of the bacterial cells. The mixture was incubated for 15 minutes on ice with vortex cycles of 10 seconds every 3 minutes. Subsequently, the elution product was centrifuged at 12,000 xg for 10 min to recover the supernatant. Neutralization buffer (1 M Tris-HCl at pH 9.0) was added to this phase at a 1: 1 (v/v) ratio and kept on ice for 30 min. Finally, the mixture was centrifuged, and the amount of IgA1 and IgA2 immunoglobulins recuperated in the supernatant was determined by ELISA. The remaining supernatant was used for the carbohydrate microarrays assay. To identify IgA subclasses reactivity against bacteria carbohydrates, we used Glycan Array 100 for 8 sample kits (Cat# GA-Glycan-100-1 RayBiotech^®^ Norcross GA, USA). Slides were rinsed with PBS and blocked with 1% BSA in PBST containing 0.025% NaN_3_ at room temperature for 30 min. Each subarray was stained with 40 µL of each supernatant sample in 1:9 dilution with PBST containing 0.05% NaN_3_ and 1% BSA. The slide was incubated in a humidified chamber at room temperature for 90 min. The slide was washed five times with PBST and then incubated with 40 µL of secondary mouse antibody anti-human IgA2 coupled with FITC (Cat# 9140-02 SouthernBiotech^®^, Birmingham, AL, USA) dilution 1:2500. The slide was incubated in a humidified chamber with light protection at room temperature for 30 min and washed five times, as previously described. Fluorescence signals were detected with Chemidoc™ XRS+ System Molecular Imager Gel (BioRad^®^).

### Semi-Quantification of IgA Bound to Bacteria by Western Blot

A volume of colostrum fractions (Total, IgA1+, and IgA2+ microbiota) was adjusted at 10,000 bacteria per mL. We used the purified human protein IgA1 (Cat# ab91020 Abcam^®^) and human protein IgA2 (Cat# ab91021 Abcam^®^) diluted in serial double dilutions in PBS for calibration curves. All bacterial fractions were lysed for 30 min at 4°C with RIPA buffer (20 mM Tris-HCl pH 7.5, 150 mM NaCl, 1 mM Na_2_EDTA, 1 mM EGTA, 1% NP-40, 1% sodium deoxycholate, 1 mM Na_3_VO_4_, and 1 ug/mL leupeptin). The lysates were centrifuged at 4°C for 15 min at 12,000 xg. The supernatants were separated, and proteins were quantified by the Lowry method. Samples were mixed with NuPAGE Sample Reducing Agent 10x (Cat# NP0004 Life Technologies^®^ Thermo Fisher Scientific^®^). The samples were separated by electrophoresis in 12% Tris–HCl gels (Cat# 3450009 Bio-Rad^®^) at 80 V for 90 min and transferred to nitrocellulose membranes 0.45 µm (Cat# 1620115 Bio-Rad^®^) at 100 V for 120 min. Membranes were blocked for 1 hour in 5% non-fat dry milk (Cat# 9999S Cell Signaling Technology^®^ Danvers MA, USA) in PBST at 4°C. After three wash cycles with Tris Buffered Saline with Tween 20^®^ (TBST) (Cat# 91414, Sigma-Aldrich^®^), the membrane was incubated overnight at 4°C with biotin-coupled mouse antibody anti-human IgA1 (Cat# ab99796, Abcam^®^) at 1:8,000 and a biotin-coupled anti-human IgA2 (Cat# ab128731, Abcam^®^) at 1:10,000. After that, 1: 2,000 dilutions of HRP-conjugated streptavidin (Cat# ab7403 Abcam^®^) were added to the membranes and incubated for 1.5 h at room temperature (as described above). Subsequently, labeled proteins were visualized using the Clarity™ Western ECL Substrate kit (Cat# 170-5061 BioRad^®^). The emitted chemiluminescent signal was detected with a Chemidoc™ XRS+ System Molecular Imager Gel (BioRad^®^), and ImageJ^®^ software (ImageJ) was used to estimate the relative amounts of protein.

### Serine Hydroxymethyltransferase (SHMT) Western Blotting

Bacterial suspensions IgA1+ and IgA2+ from human colostrum were treated as previously mentioned to obtain soluble IgA1 and IgA2 supernatants. They were mixed with NuPAGE Sample Reducing Agent 10x (Cat# NP0004 Life Technologies^®^ Thermo Fisher Scientific^®^). Three control lanes were added: positive control, composed by recombinant bacterial SHMT (#Cat LS-G78730-20 LS Bio^®^, Seattle WA, USA), negative control, composed by purified bacterial Glyceraldehyde 3-phosphate dehydrogenase (GAPDH) (#Cat NATE-1635 Creative Enzymes^®^, Shirley NY, USA), and colostrum bacteria non-coated with IgA subclasses (IgA-). Two hundred micrograms of proteins were loaded into each lane, separated in 10% polyacrylamide electrophoresis gels (Cat# 3450009 Bio-Rad^®^) at 80 V for 90 minutes transferred to nitrocellulose membranes (Cat# 1620115 Bio-Rad^®^) at 100 V for 120 min. Membranes were blocked in 5% nonfat dry milk (Cat# 9999S Cell Signaling Technology^®^ Danvers MA, USA) in TBST. After three washes with PBST, 2 µg/mL of anti-SHMT mouse monoclonal IgA antibody (clone W27) was added and incubated overnight at 4°C, as previously reported (44). The following day, the membrane was washed, and a mixture of HRP-goat anti-mouse IgA (Cat# 1040-05 Southern Biotech^®^) at 1: 8,000 dilution, and HRP-goat anti-bacterial GAPDH (Cat# G8140-13H United States Biological^®^, Salem MA, USA) at 1: 10,000 were added and incubated two hours at room temperature. Finally, the membrane was washed, and signals were visualized using the Clarity™ Western ECL Substrate kit (Cat# 170-5061 BioRad^®^) and detected in ChemidocTM XRS+ System Molecular Imager Gel (BioRad^®^) and ImageJ^®^ software.

### Statistical Analysis

Data were reported as mean ± standard deviation (SD) or frequencies and percentages (%). Two tailed Student’s t-test, Mann-Whitney U test or Welch´s t test were assessed to compare groups using SPSS v23.0 software (SPSS, Inc). ANOSIM and Adonis were used for category comparisons of phylogenetic distance matrices (UniFrac). Linear regression was used to find the relationship between microbiota diversity as the dependent variable and maternal and neonatal age included as covariates; p ≤ 0.05 was considered statistically significant. The Benjamini−Hochberg (BH) correction method was used to estimate the false discovery rate (FDR) and filter the data where a q-value ≤ of 0.05 was considered statistically significant.

## Results

### IgA Subclasses Differentially Recognize a Group of Bacteria in Human Colostrum

**Figure 1:**
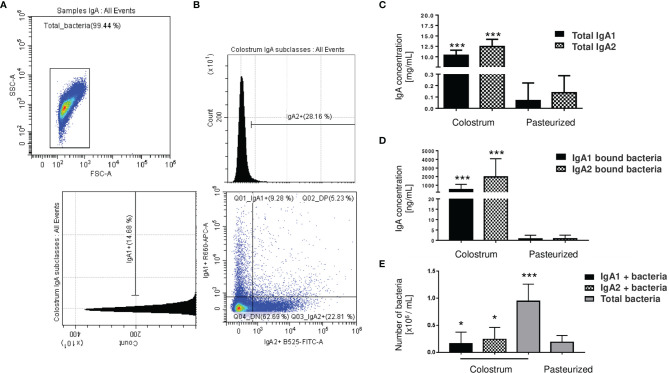
Microbiota from the human colostrum is differentially coated with IgA subclasses. **(A)** Staining profiles of bacteria in human colostrum characterized by flow cytometry. **(B)** Analysis of bacterial populations by their associations with IgA1 (APC Cy7) and IgA2 (FITC) in human colostrum. Plots are representative of thirty-six biological samples analyzed. **(C)** Quantification of free IgA subclasses in colostrum, titled “Colostrum,” and pasteurized milk, titled “Pasteurized”: by ELISA. Data are expressed in milligrams of IgA subclasses per milliliter of the sample (mg/mL). **(D)** Semi-quantification by Western blot of IgA subclasses’ levels bound to bacteria, data are expressed in nanograms of IgA subclasses per milliliter of the sample (ng/mL). IgA1 (black bars) and IgA2 levels (filled bars) from Colostrum and Pasteurized milk. **(E)** Numbers of bacteria determined by flow cytometry. Data are expressed in millions of bacteria per milliliter of the sample (10^6^ bacteria/mL). IgA1+ (black bars), IgA2+ (filled bars) and total bacteria (gray bars). Bars indicate the mean ± SD of triplicates from thirty-six “Colostrum” and twenty-eight “Pasteurized” milk samples. Statistical analysis was performed using the Mann-Whitney U test, comparing each IgA subclasses level with the pasteurized milk sample as a control. *p < 0.05 and ***p < 0.001.

We analyzed percentages of bacteria coated with IgA subclasses from total bacteria present in human colostrum samples (**Figures 1A, B**). Data showed that IgA1 is associated with 9.67% ± 5.48 of the total bacteria present in colostrum, 21.40% ± 7.36 are associated with IgA2, and 5.09% ± 3.07 are coated with both subclasses (**Figure 1B**); demonstrating that IgA subclasses have a selective recognition with colostrum bacteria. To evaluate how the colostrum microbiota coated with IgA affects microbiota establishment in neonate’s intestine, we characterized and compared IgA subclasses associated with bacteria in maternal colostrum (Colostrum) *versus* pasteurized milk (Pasteurized) samples (**Figures 1C–E**). IgA2 has slightly higher concentrations than IgA1 in colostrum (**Figure 1C**). At the same time, IgA2 is more frequently associated with bacterial surfaces (**Figure 1D**). In comparison, IgA subclasses in pasteurized milk samples were significantly reduced. Milk pasteurization denatures antibodies affecting concentration and ability to recognize antigen (45). We observed fewer bacteria with IgA associated on their surfaces in pasteurized milk samples (**Figure 1D**) and, in general, a significantly reduced number of bacteria present in these samples (**Figure 1E**). Consequently, IgA1+ and IgA2+ bacteria fractions in the pasteurized milk samples were not detected. These results demonstrate that fresh, non-pasteurized milk is the source of bacteria coated with IgA.

### IgA Subclasses in Colostrum Have a Selective Recognition for Some Groups of Bacteria From the Microbiota

**Figure 2:**
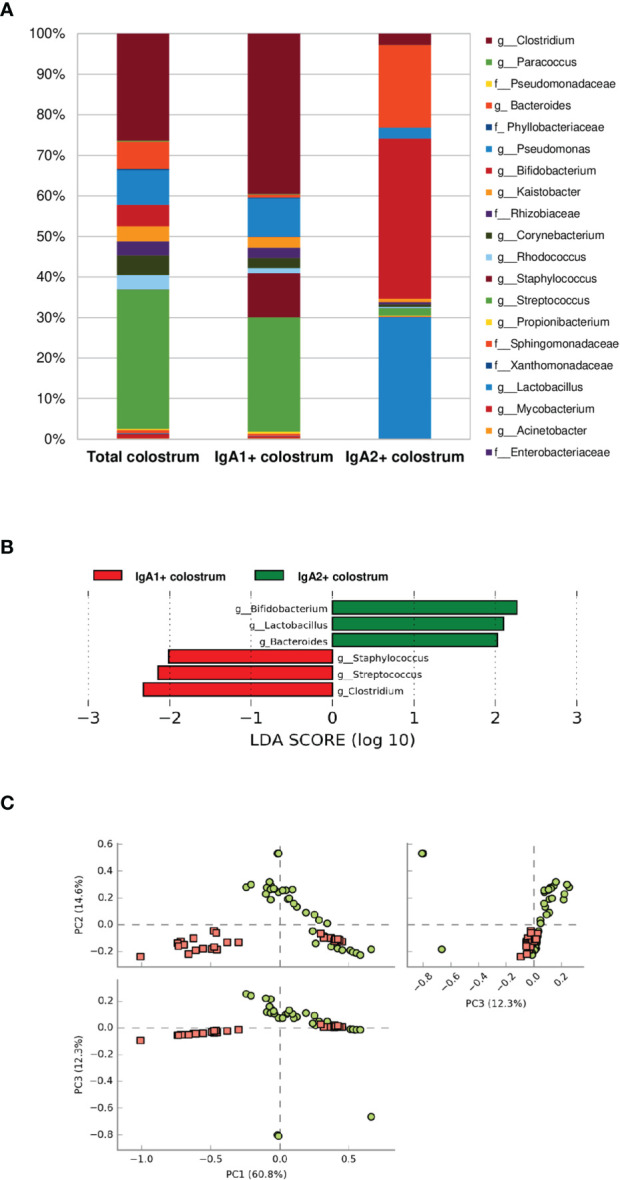
Some bacteria have divergent recognition between IgA subclasses in the human colostrum. **(A)** Relative abundance of predominant bacterial taxa in colostrum samples. Abundance of each families/genera in IgA fractions (Total bacteria in colostrum = Total colostrum, bacteria coated with IgA1= IgA1+ colostrum, and bacteria coated with IgA2 = IgA2+ colostrum). IgA1+ fraction is plotted as red dots and IgA2+ fraction as green dots. Previous graphs represent data from thirty-six “Total bacteria,” thirty “IgA1+ colostrum,” and thirty-three “IgA2+ colostrum” samples. Both groups significantly differed according to the ANOSIM similarity test (R^2^ = 0.289, p = 0.001) and Adonis statistical test (R^2^ = 0.949, p = 0.001). **(B)** LEfSe analysis of the most representative bacterial genera in colostrum by IgA1 and IgA2. Linear discriminant analysis (LDA) effect size (LEfSe) compares differential abundant bacterial taxa between IgA fractions. Horizontal bars represent each taxon’s effect size: green indicates taxa enriched in the IgA2+ fraction group, and red indicates taxa enriched IgA1+ fraction. LDA score cutoff of 2.0 was used to discriminate bacterial taxon. **(C)** Beta diversity analysis. Two-dimensional scatterplots were generated using PCoA based on the unweighted UniFrac distance metric. PC3 *vs* PC2, PC1 *vs* PC2, and PC1 *vs* PC3.

According to their association with IgA subclasses, bacterial microbiota relative abundances were determined at the genus and family level in the fractions. Twenty bacterial genera were predominantly in colostrum, including *Clostridium, Bacteroides, Pseudomonas, Bifidobacterium, Kaistobacter, Corynebacterium*, the *Enterobacteriaceae* family (**Figure 2A**). As previously mentioned, bacteria fractions showed distinctive genera between IgA+ subclasses fractions in human colostrum (**Figure 2A**). This sharp divergence in the composition between IgA subclasses fractions composition was confirmed by LEfSe analysis (**Figure 2B**). LEfSe (Linear discriminant analysis effect size) algorithm was used to identify significant taxonomic differences in the microbiota among the groups of samples studied, like previously reported (46). For analysis between the IgA subclass fractions in colostrum samples, it was found that six bacterial genera, including *Clostridium, Streptococcus*, and *Staphylococcus*, were recognized by IgA1. In contrast, *Bifidobacterium, Pseudomonas, Lactobacillus*, and *Bacteroides* were recognized by IgA2. The distribution between fractions was significantly different (**Figure 2B**). PCoA analysis determined that, although individual samples share components between IgA fractions, most of them presented differences depending on IgA subclass recognition (**Figure 2C**). These data demonstrate a divergent recognition of microbiota by IgA subclasses in human colostrum.

### A Percentage of Recovered Bacteria in Stool Samples From Neonates Fed Exclusively With Colostrum Was Uniquely Associated With IgA2

**Figure 3:**
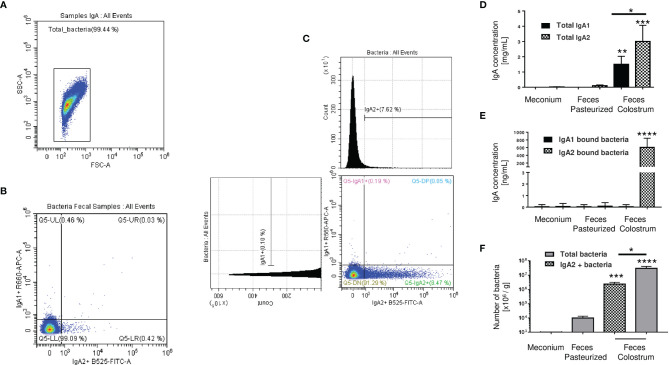
IgA2 is predominantly found coating bacteria in newborn feces of breastfed children. **(A)** Dot blot of total bacteria in neonate’s fecal samples. **(B)** Bacteria found in meconium characterized by flow cytometry. **(C)** Bacterial populations coated with IgA subclasses in stool samples. Plots are representative of five pool meconium **(B)** and nineteen feces’ samples **(C)**, obtained after three days of colostrum-fed newborn children. **(D)** Quantification by ELISA of IgA subclasses in “Meconium,” a stool sample from children fed with pasteurized milk, titled “Feces Pasteurized,” or children fed with maternal colostrum, titled “Feces Colostrum”; data are expressed in milligrams of IgA per gram of fecal sample (mg/g). **(E)** Semi-quantification by Western blot of IgA subclasses’ levels bound to bacteria; data are expressed in nanograms of IgA per gram of fecal sample (ng/g). IgA1 (black bars) and IgA2 levels (filled bars). **(F)** Number of bacteria determined by flow cytometry; data are expressed in millions of bacteria per gram of fecal sample (10^6^ bacteria/g). IgA2+ (filled bars) and total bacteria (gray bars). Bars indicate the mean ± SD of triplicates from five “Meconium,” twenty-seven “Feces Pasteurized,” and nineteen “Feces Colostrum” samples. Statistical analysis was performed using the Mann-Whitney U test between IgA subclasses in the same sample and comparing each level with feces pasteurized levels as control. *p < 0.05, **p < 0.01, ***p < 0.001 and ****p < 0.0001.

The next step was to evaluate the microbiota present in newborn meconium and stool samples according to its association with IgA subclasses (**Figures 3A–C**). As expected, bacteria coated with the IgA subclasses were not found in meconium samples (before breastfeeding) (**Figures 3A, B**). Breastfeed infants had the highest levels of IgA subclasses (**Figure 3D**), IgA-associated microbiota (**Figure 3E**), and bacterial quantity (**Figure 3F**) compared to pasteurized milk-fed infants (**Figures 3D–F**). Free IgA1 concentration in feces decreased in proportion to what was found in colostrum; however, IgA1-associated microbiota was neither found in breastfed-infants (**Figure 3C**) nor pasteurized milk fed samples (**Figures 3E, F**). Perhaps the presence of IgA1-specific bacterial proteases in addition to intestinal lumen conditions may explain this reduction (47, 48). IgA2 would be more resistant to these conditions. We developed a kinetic enzyme assay to explain the observed differences. The data indicated that only 20 minutes of exposition to the bacteria from either colostrum or feces is enough to decrease 40% ± 1.21 the amount of IgA1 (**Supplementary Figure 5**). A possible explanation is that IgA1 from the colostrum is degraded faster by bacteria and enzymes in the newborn intestine. For that reason, it is not detected coating the bacteria recovered from the feces of breastfed infants. Thus, these results suggest that a percentage of the recovered bacteria were associated solely with IgA2 in stool samples from neonates fed exclusively with colostrum during the first three days of life (**Figure 3F**). In contrast, no bacteria coated with IgA were found neither in pasteurized milk-fed infants nor in meconium samples.

### IgA2+ Microbiota Compositions Are Similar Between Colostrum and Fecal Samples in Neonates

**Figure 4:**
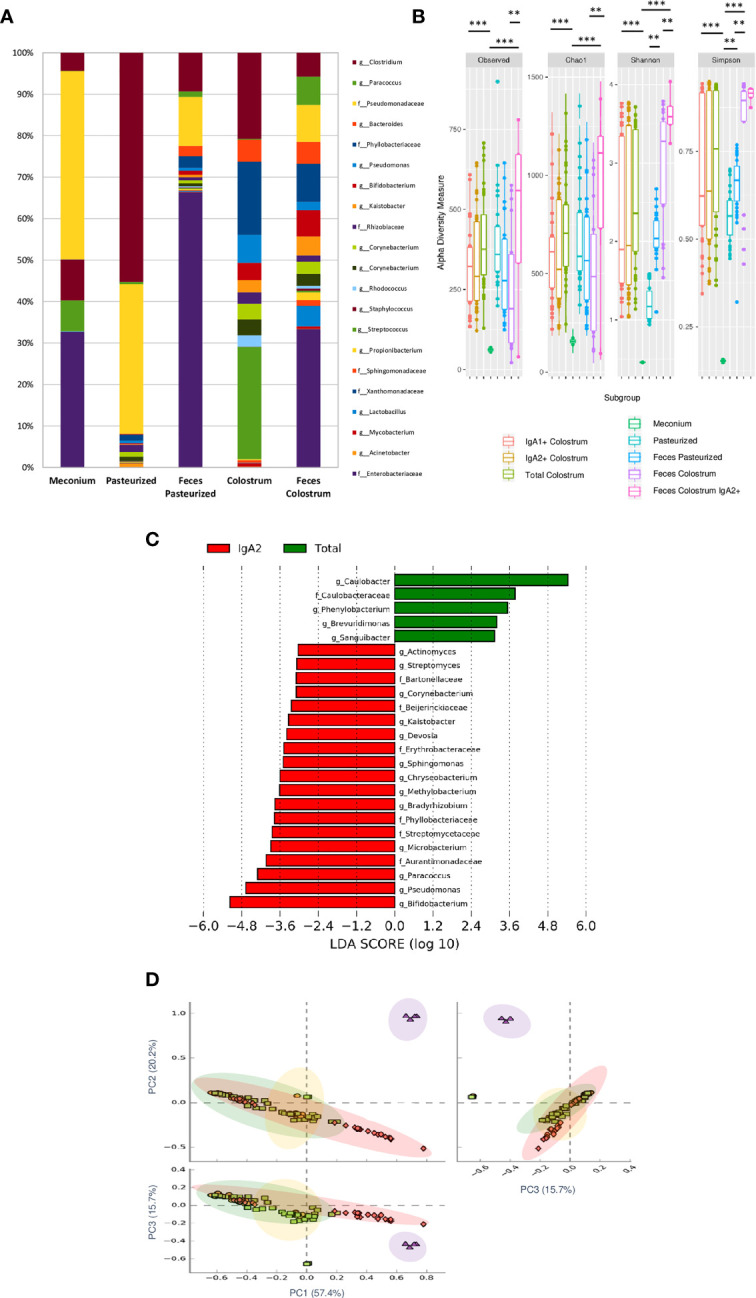
IgA2+ microbiota is present in fecal neonate’s samples. **(A)** Relative abundance of predominant bacterial taxa in stool samples (Meconium, neonates fed with pasteurized milk= “Pasteurized,” neonates fed with pasteurized milk= “Feces Pasteurized” and neonates fed with colostrum= “Feces Colostrum”; during first three days of life). The abundance of each family/genera in IgA fractions was compared between groups using a parametric test for paired samples followed by BH correction. **(B)** Alpha diversity based in observed number of species (p < 0.001), Chao1 (p < 0.001), Shannon (p < 0.001) and Simpson (p = 0.006) indexes. Mann-Whitney U test was used to find significant differences. Alpha diversity based on observed number species (p < 0.001), Chao1 (p < 0.001), Shannon (p < 0.001) and Simpson (p < 0.005) indexes. Mann–Whitney U test was used to find significant differences **p < 0.01, ***p < 0.001. **(C)** LEfSe analysis of the most representative bacterial genera in stool samples. Linear discriminant analysis (LDA) effect size (LEfSe) comparison of differentially abundant bacterial taxa among free fecal bacteria (“Total”) and IgA2+ fecal bacteria (“IgA2”); from neonates whose were fed with colostrum during the first three days of life. Horizontal bars represent each taxon’s effect size: red indicates taxa enriched in the IgA2+ fraction group, and green indicates free taxa enriched group. LDA score cutoff of 2.4 was used to discriminate bacterial taxon. Previous graphs represent data from five “Meconium”, twenty-eight “Pasteurized milk”, twenty-seven “Feces pasteurized”, thirty-six “Total colostrum”, thirty “IgA1+ colostrum”, thirty-three “IgA2+ colostrum”, nineteen “Feces Colostrum” and five “IgA2+ Feces” samples. **(D)** Two dimensional scatterplots were generated using PCoA based on the unweighted UniFrac distance metric. PC3 *vs* PC2, PC1 *vs* PC2, and PC1 *vs* PC3. Both groups significantly differed according to the ANOSIM similarity test (R^2^ = 0.289, p = 0.001) and Adonis statistical test (R^2^ = 0.949, p = 0.001). Total bacteria from colostrum samples are represented in red diamonds, IgA2+ fecal bacteria from neonates fed with colostrum in green squares, total bacteria from neonates fed colostrum in yellow circles, and total bacteria from pasteurized milk in purple triangles.

Our results found that meconium contains a higher abundance of the genera *Bifidobacterium, Pseudomonas, Staphylococcus, Clostridium, Streptococcus*, and the *Enterobacter* family. In contrast, pasteurized milk samples showed a greater abundance of *Clostridium* and *Pseudomonas* genera even after removing contaminant DNA sequence. These taxa have been commonly reported as contaminants in milk samples (42). However, like other previous works, we identified an enrichment of these genera after pasteurized process in human milk samples (7). Finally, the bacterial composition in stool samples of children fed with colostrum (*Clostridium, Pseudomonas, Bacteroides, Bifidobacterium, Kaistobacter, Lactobacillus*, and *Enterobacteriaceae* family) showed significant differences in comparison with those fed with pasteurized milk (*Clostridium, Pseudomonas*, and *Enterobacteriaceae* family) (**Figure 4A**). Observed OTUs, Chao1, Shannon, and Simpson indexes were used to analyze Alpha diversity within each sample. Total bacterial fraction showed higher bacterial richness and diversity than IgA subclasses (p=0.03 for the nonparametric Wilcoxon rank test) (**Figure 4B**). Regarding the Shannon index, higher values were found in stool samples of infants fed with breast milk and colostrum than pasteurized milk-fed infants and meconium samples (**Figure 4B**). As expected, meconium and pasteurized milk samples had the highest dominance indexes. For the rest of the samples, we did not find a statistically significant difference.

For the bacteria coated with IgA2, the result showed 24 taxonomic groups significantly different, 19 for IgA2+ and 5 for free bacteria in feces (**Figure 4C**). However, a comparison between IgA2 fraction samples from colostrum and breastfed children’s, IgA2+ bacterial feces demonstrated they had shared some bacterial composition. According to PCoA analysis, the data showed that fecal bacteria composition from three-day-old breastfeeding neonates is more like the fraction of IgA2+ bacteria in colostrum than samples from children fed with pasteurized milk (**Figure 4D**). This result was corroborated with beta diversity analysis compared with other analyses like weighted UniFrac and Bray-Curtis distances. To estimate what portion of the fecal microbiota could come from colostrum, we used Source Tracker analysis. The analysis suggested that 70% of total intestinal microbiota had its origin in the colostrum (**Supplementary Figure 6A**). This microbiota is enriched of *Clostridium, Bifidobacterium*, and *Lactobacillus* genera (**Supplementary Figure 6B**). The remaining 30% of the microbiota is characterized by bacteria from other sources like the human perianal zone (49). When we, specifically, estimate the probable origin of fecal IgA2+ bacteria fraction, we found that more than 60% had its origin in IgA2+ colostral bacteria fraction (**Supplementary Figure 6C**). This bioinformatics analysis strongly suggest that colostrum is a source of neonate bacteria, and the association with IgA2 could provide them an advantage during microbiota establishment in the newborn intestine.

### IgA2+ Bacteria in Feces From Breastfed Infants Showed Enrichment of Some Predicted Metabolic Pathways

**Figure 5:**
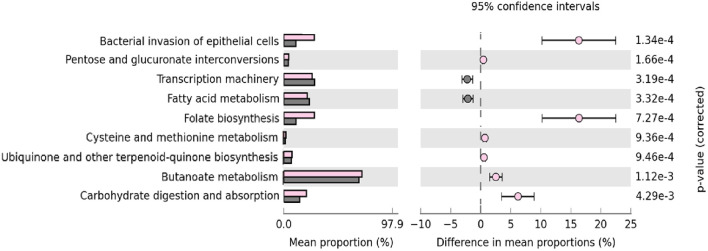
Microbial metabolic pathways among IgA2+ bacteria are enriched in intestine adherence and active carbohydrate metabolism. GO analysis of the most representative metabolic pathways in predicting functional microbial metabolic pathways using PICRUSt analysis (KEGG level three). The abundance of nine statistically significant metabolic pathways between five IgA2+ fecal bacteria samples from neonates fed with human colostrum for three days, in pink; and twenty-seven IgA- fecal bacteria samples from children fed with pasteurized milk, in gray. All values are represented as mean ± SD.

Finally, we used PICRUSt to analyze which predicted metabolic pathways associates to the relative abundances of bacteria present in the samples. This analysis also examines whether there are disparities in bacterial composition between IgA2+ fractions of colostrum and feces of neonates exclusively fed with breast milk. PICRUSt analysis identified nine functional metabolic pathways and showed that they have functional profiles despite their different inter-and intra-individual variation (alpha and beta diversity) of the neonatal intestinal microbiota and colostrum. In general, IgA2+ bacteria in feces from breastfed infants showed a significantly increased abundance of metabolic pathways. These pathways were overrepresented in feces samples from infants fed with colostrum (p <0.04) compared to formula feeding. The bacterial invasion of epithelial cells, folate biosynthesis, and carbohydrate metabolism had the most significant difference (p = 1.34e^-4^). In the same way, transcription machinery, fatty acid metabolism, and pentose-glucuronate interconversions metabolic pathways were underrepresented in the same fractions (6.76e^-3^) (**Figure 5**). All these data suggest that IgA2 association could help shape microbiota on neonate, providing an advantage to associate specific genera enriched with folate biosynthesis and carbohydrate metabolism to establish in the mucosal intestine.

### IgA2 Recognizes SHMT and Some Glycans Arrangements on Bacteria Surfaces

Since our results indicated a bacterial enrichment of metabolic pathways associated with folate and carbohydrates synthesis, we decided to determine, as previously published (44, 50), possible antigens recognized by IgA subclasses on bacterial surfaces. The results showed IgA2 binding to carbohydrates like N-acetylglucosamine (D-GlcNAc), D-Mannose (DMan), and alpha-D-Mannose (a-D-Map) (**Supplementary Figure 7A**). In addition, colostrum IgA subclasses recognized SHMT, which is present in bacterial extracts of both isolated fractions (IgA1+ and IgA2+) (**Supplementary Figure 7B**). Thus, we suggest that IgA2 may interact with colostrum bacteria by recognizing common antigens among different bacterial genera under these experimental conditions. The results agree with a previous report on mice intestines (50), however, as authors discussed, microbiota recognition by IgA is more complex and dynamic process and probably is related to more variables that we did not consider in this study.

## Discussion

This work is based on bacterial DNA sequencing of the IgA2-colostrum and IgA2-neonatal fecal microbiota, to provide information about the dynamic and association of colostrum IgA2 in fecal microbiota during the postnatal stage. We present data about the IgA2 bacterial coating in the neonatal feces, through the carbohydrate recognition by the maternal IgA2 from colostrum, being these bacteria one of the primary bacterial sources for the neonate’s microbiota.

We described that meconium microbiota was characterized by the *Enterobacteriaceae* family and *Pseudomonas* genera, with enrichment of *Clostridium, Staphylococcus*, and *Streptococcus* genera whose origin is still under discussion (51). During vaginal birth, the neonate is colonized by maternal microbiota from the urogenital tract and perianal zone, characterized by the genera *Clostridium, Pseudomonas, Bifidobacterium, Lactobacillus*, and the *Enterobacteriaceae* family.

The mother provides, through colostrum feeding during the first three days, bacteria of the genera *Lactobacillus, Bacteroides, Pseudomonas, Bifidobacterium*, and *Propionibacterium* coated with IgA2 (IgA2+). According to previous reports, breastfeeding could cooperate to shape a neonatal intestinal microbiota increased in different genera from the *Enterobacteriaceae* family and *Pseudomonas, Bacteroides, Bifidobacterium, Lactobacillus*, and *Propionibacterium;* curiously, these we found these genera were coated with IgA2 (IgA2+). Meanwhile, *Paracoccus, Phyllobacteriaceae*, and *Clostridium* appears to remain without IgA association (IgA2-). This observation was corroborated when we compared our data with fecal samples from neonates fed with pasteurized milk. This milk had low bacteria content and microbiota dominated by bacteria that can resist pasteurization processes like *Clostridium* and *Pseudomonas* (52). This low-quality milk shapes an intestinal microbiota dominated mainly by *Clostridium, Pseudomonas*, and *Enterobacteriaceae*, associated with problems in neonate’s health (53). These results suggest that maternal colostrum provides and helps to modulate microbiota in neonates. However more studies are needed.

In accord with our results, the predominance of IgA2 in fecal samples could be a consequence of IgA1 degradation by bacterial enzymatic activity. Moreover, compared to the adult fecal sample, colostrum contains a lower microbiota density, perhaps promoting a more selective IgA association with a wide variety of immunogenic antigens on bacteria surfaces (53).

Like Huus et al. reported, we found that IgA2 recognizes some glycans arrangements on bacteria surfaces, for example, *Lactobacillus* genera (50, 54). However, this recognition is dependent on different variables, suggesting that the interaction of IgA2 with microbiota is more complex (55). In the present work, we used a carbohydrate microarray to describe the glycans recognized by IgA2. The results obtained may explain how is the recognition of bacteria surfaces. However, IgA bound to microbiota cannot only rely on IgA specificity or affinity; there are more parameters involving the regulation of the expression of carbohydrates by bacteria (50). It seems, however, that IgA2 may recognize through medium/low-affinity binding to T-independent antigens such as glycans. Thus, IgA2 associated with the bacterial surface may give this group of bacteria some advantage in binding to the mucus layer, driving microbial cores on intestine surfaces (56).

Furthermore, association with IgA may promote a tolerogenic response against these bacteria (57, 58). Finally, IgA may also generate niches with commensal bacteria communities in the intestine (59). Thus, IgA may control the arrival of non-pathogenic microbiota (60), improving their enrichment in feces (61, 62). But, as mentioned above, the problem of microbiota recognition and colonization is more complex than initially thought, so this is an area of opportunity for future research.

Fecal IgA2+ microbiota overrepresented carbohydrate consumption and bacterial invasion pathways; meanwhile, a slow-growth ratio is related to machinery transcription, fatty acid metabolism, and pentose-glucuronate interconversions. *Bifidobacterium, Pseudomonas, Lactobacillus*, and *Paracoccus* genera represent metabolic pathways related to the latency growth phase during the first three days (63); consuming carbohydrates and generate physicochemical conditions in small microbial niches (64). After that, these bacteria should enter to logarithmic growth phase after five days of adaptation, increasing transcription, DNA, and fatty acid synthesis.

On the other hand, *Bifidobacterium, Pseudomonas, Paracoccus*, and bacteria from the *Enterobacteria* families were coated with IgA2 in stool samples only after three days of feeding with colostrum. These data suggest that IgA2 recognizes and could join with other bacterial genera in the newborn gut. Through this interaction, soluble maternal IgA2 could help to regulate the composition of the microbiota *in situ*.

Some bacterial genera such as *Caulobacter, Phenylobacterium*, and *Brevundimonas*, found in the newborn’s feces, were not coated with IgA2. Then other subsequent genera found in the infant’s feces like *Paracoccus*, *Clostridium*, and the *Phyllobacteriaceae* family, could take advantage of the IgA2+ bacteria’s metabolic activity. This result suggests a much more dynamic interaction and metabolic activity between the different bacterial genera that are present in feces in the first moments of life (65, 66). However, more studies are needed to understand this phenomenon.

According to our data, the most widely represented metabolic pathways are folate synthesis and carbohydrate metabolism. Furthermore, Okai S et al. determined that, in the mouse intestine, bacteria that expressed SHMT enzyme, which regulates folate metabolism, were recognized by high-affinity IgA. This interaction shapes microbiota composition in mice (44); however, this issue is not well understood in humans.

Among the main limitations of this work are that feces do not necessarily represent the entire intestinal microbiota composition. Feces have become the reference of most bacteria studies because of is a non-invasive methodology, and sampling is easy and convenient. Previous studies in animal models had demonstrated an essential variation between samples (67). Perhaps, fecal samples could be more like intestinal microbiota composition in neonates because before delivery is a relatively sterile mucosa and at colon level (68). More studies are necessary to clarify if feces analysis truly represents intestinal microbiota in the newborn. Of course, breastfeeding is only one of the many microbiota sources during the first days of life.

Although we have no definitive evidence to demonstrate that IgA2-coated bacteria in the colostrum can transit through the neonate’s intestine and colonize the intestine, the evidence suggests that at least IgA2 may play a role in the intestinal bacterial composition. Newborn children do not produce IgA, then all IgA found in feces after three days of colostrum breastfeeding must come from the mother. Infants fed with pasteurized milk lack IgA in their feces because pasteurization denatures IgA. Then, as different animal models have demonstrated, efficient transit of maternal milk bacteria may provide some advantage to groups of bacteria recognized by IgA (16). However, more studies are necessary to explore the role of human IgA subclasses in the intestine microbiota during the first days of life.

Another limitation of this work is that meconium samples analysis diminished individuality. We decided to pool the samples to have a broad vision of the possible composition of intestinal microbiota before breastfeeding and to secure sufficient bacterial quantity for the subsequent experiments. We use five meconium pooled data to determine the first microbiota immediately after delivery and monitor maternal IgA-coated microbiota’s effect on bacteria composition in the neonatal feces. Because of technical limitations in the number of bacteria (low biomass samples), our primary focus on the present study is comparing the effect on infants’ microbiota composition after three days with colostrum breastfeeding *versus* infants fed with pasteurized milk. In addition, our analysis was limited for the comparison of beta diversity among all colostrum and fecal fraction groups and no by each neonate-mother binomial (68). Therefore, the use of pooled samples implicates those definitive conclusions, and discussions must be taken with caution. However, we believe our work provides avenues for further, more detailed research. Since pooled samples, mainly in low biomass samples, have been considered valid in bacteria composition analysis (68). As in the publication referred, we used just the statistical analysis they mentioned as valid to compare pooled samples (68).

Finally, it is important to consider that, the applied methodology for the bacterial identification in this work (sequence amplicon V3-16S rRNA gene by PGM) did not provide taxonomic resolution at species level. But despite that, this work provides important information about the general IgA2 coated-bacterial community in both niches (colostrum and infant gut microbiota). To a better understanding, other analysis is required, as specific bacterial isolation and WGS to perform either genome comparison, MLST analysis, or metagenome analysis (69). We hope to perform a different, more informative approach in future work.

This work was focused on IgA2 microbiota in colostrum and their similarity with fecal neonate bacterial composition. However, IgA1 could have a more active function defense against pathogens in neonate intestine. Thus, future studies in animal models are necessary due to the experimental limitation described above.

The whole work is not just a mere bacterial microbiota characterization. The neonate acquires the bacterial microbiota from the mother’s milk, and this microbiota is already differentially recognized by IgA, and definitively it strongly suggests an influence in the composition of the gut microbiota in the neonate. At the same time, this work contributes to the knowledge of human milk and neonatal stool microbiota in a healthy population and supports the idea of mother-neonate transmission through exclusive breastfeeding.

## Conclusion

The composition of the IgA2 microbiota is shared between colostrum and fecal samples from the newborn. These results could explain how the design of the neonatal microbiota is shaped in the intestine. The results also could provide a model to study the relative contribution of both IgA and bacteria present in the colostrum. In addition, groups of bacteria coated with IgA2 show metabolic profiles that could induce and control the posterior microbiota, suggesting an active role for this IgA2-microbiota association beyond the first days of life. This IgA2-microbiota interaction could give these bacteria an evolutionary advantage to colonize the neonatal intestine, at least during the first three days of life.

## Data Availability Statement

The datasets presented in this study can be found in online repositories. The names of the repository/repositories and accession number(s) can be found below: https://www.ncbi.nlm.nih.gov/, prjna707069.

## Ethics Statement 

The Research and Ethics Reviewing Board approved the study protocol of Protocols from HR 1° Oct (with number 090201/14.1/086/2017).

## Author Contributions

ES-S performed experiments, analyzed the data, interpreted the results, and wrote the manuscript. KC-C participated in molecular biology experiments and headed bioinformatics analysis during all projects. HG-A and MB-C contributed to collecting samples, collecting clinical information, and participating in flow cytometry assays. VC-V supervised the sampling process, provided clinical data, and registered the protocol at the Hospital. AP-E was responsible for the massive sequencing process. JG-M and LS-A conceived and designed the study, wrote, and revised the manuscript’s final version. All authors contributed to the article and approved the submitted version.

## Funding

This research was supported by grants from Consejo Nacional de Ciencia y Tecnología (CONACYT), Mexico (PDCPN 2015/900) to LS-A, CONACyT 163235 INFR-2011-01 granted to JGM, and SEP-Cinvestav funding (174) to LS-A and JG-M. ES-S (706312) and KC-C (777953), received a scholarship from CONACYT.

## Conflict of Interest

The authors declare that the research was conducted in the absence of any commercial or financial relationships that could be construed as a potential conflict of interest.

## Publisher’s Note

All claims expressed in this article are solely those of the authors and do not necessarily represent those of their affiliated organizations, or those of the publisher, the editors and the reviewers. Any product that may be evaluated in this article, or claim that may be made by its manufacturer, is not guaranteed or endorsed by the publisher.

## Supplementary Material

Supplementary Figure 1 Controls for flow cytometry bacterial detection, staining, and magnetic fractionation. **(A)** Size and complexity profile of total bacteria from samples (top). Dot blot of *E coli* used as control (middle) and viability assay with Syto9 kit^®^ in bacteria samples (bottom). These parameters were used to adjust gates in size and complexity events into bacterial cells. **(B)** CD81+ staining control to exclude possible contamination with exosomes. **(C)** Staining of samples with antihuman IgE as an isotype control, then streptavidin-APC Cy7 (left) and streptavidin-FITC (right). **(D)** Polystyrene microbeads coated with purified human IgA1 (above) or IgA2 (below); stained with mouse anti-human IgA1 (right) or mouse anti-human IgA2 (left) to show the specificity of the reagents used during bacterial detection, staining, and magnetic fractionation. Figures include the percentage of positive events for each condition. Plots are representative of thirty-six biological samples analyzed.

Supplementary Figure 2 Analysis of bacterial populations in colostrum by their associations with IgA1 (APC Cy7) and IgA2 (FITC) in human colostrum, before (above) and after negative (left below) and positive (right below) selections with magnetic beads separation. Plots included the percentage of purity for each condition. Positive selection products were treated for DNA extraction, library preparation, and the massive sequencing process. Plots are representative of thirty-six biological samples analyzed.

Supplementary Figure 3 Controls for low biomass samples. **(A)** Gram staining from ten meconium pool samples (above) in comparison with sampling control (below). Slides were prepared with 500 uL of resuspended samples in PBS and dry for 5 minutes before the Gram staining protocol. Magnification = 1000 x. **(B)** Meconium bacteria sample analyzed by flow cytometry (left) compared with no signal were detected in controls, including sampling material, environment, and Master mix decontamination graphs. **(C)** The amplification of ~281 bp amplicon was evaluated by electrophoretic fractionation in 2% agarose gel, comparing different six (up) and three (down) meconium samples (numbered as 1-6 and 1-3) with negative (-) and positive (+) controls. These controls consisted of decontaminated PCR mix with sterile water (negative control) and decontaminated PCR mix with DNA extracted from *the E coli* strain of reference. Controls were included in the mixed pool DNA sequences library for massive sequencing. Ion torrent did not report genetic material detected for sequencing with negative controls.

Supplementary Figure 4 Frequency patterns and decontam scores of microbial sequences from 16S rRNA gene dataset. The figure shows frequency patterns of the four most representative OTUs from the twenty-nine “TRUE” results obtained after decontam analysis. These OTUs had the highest frequency values in comparison with the other bacteria from the metadata table. Graphs compare the correlation between DNA concentrations present in the mix library *versus* frequency values from each sample. Each black dot represents a single sample.

Supplementary Figure 5 Bacterial proteases preferentially degrade IgA1. Kinetical evaluation of the stability of IgA1 (left) and IgA2 (right). IgA subclasses were incubated with IgA2+ or IgA1+ bacteria fractions from colostrum under two different temperature conditions and in the presence or absence of protease inhibitors. The amount of remaining IgA was quantified by ELISA, and the time is expressed in minutes. Each point indicates the mean ± SD of technical triplicates from eight randomly selected samples. All data are expressed in milligrams of IgA subclasses per milliliter (mg/mL). Statistical analysis was performed using the Mann-Whitney U test, comparing each condition at any given time point **p < 0.01 and ***p < 0.001.

Supplementary Figure 6 Some IgA2+ fecal microbiota founded in the newborn has its origin in the same bacteria fraction from colostrum. **(A)** Microbial source tracker analysis showing the proportion of bacteria identified in the neonatal stool classified by source. **(B)** The relative abundance of most common bacterial orders found in the neonatal stool is classified by QIIME source tracker analysis as “Other sources” and “Colostrum source.” Data are from thirty-six samples. **(C)** Microbial source tracker analysis showing the 60.7% of IgA2+ bacteria identified in the neonatal stool is classified with IgA2+ colostral origin as “IgA2+ Colostrum”. p < 0.001, Wilcoxon signed-rank test.

Supplementary Figure 7 IgA2 recognizes SHMT and some glycans on the surface of bacterial microbiota. **(A)** Bacterial carbohydrates arrangements are recognized by IgA2 from human colostrum. Results are express in normalized OD, comparing purified IgA2 signal from bacteria IgA2+ fraction *versus* IgA2 signal not associated with bacteria (soluble IgA2). The graph was made with data of triplicates from eight randomly selected samples. **(B)** Recognition analysis of IgA subclasses in colostrum to SHMT enzyme. Reactivities of six IgA2+ bacteria extracts (left) and seven IgA1+ bacteria extracts, randomly selected (right) from human colostrum, itemized with consecutive numbers. GAPDH was used as a control to confirm that comparable amounts of protein were load in each sample. Images are representative of three independent experimental repetitions.
