# Supplementary figures and images for: Maternal IgA2 Recognizes Similar Fractions of Colostrum and Fecal Neonatal Microbiota
Source: Front Immunol. 2021 Nov 4;12:712130. doi: 10.3389/fimmu.2021.712130 (PMC8601722; doi:10.3389/fimmu.2021.712130)

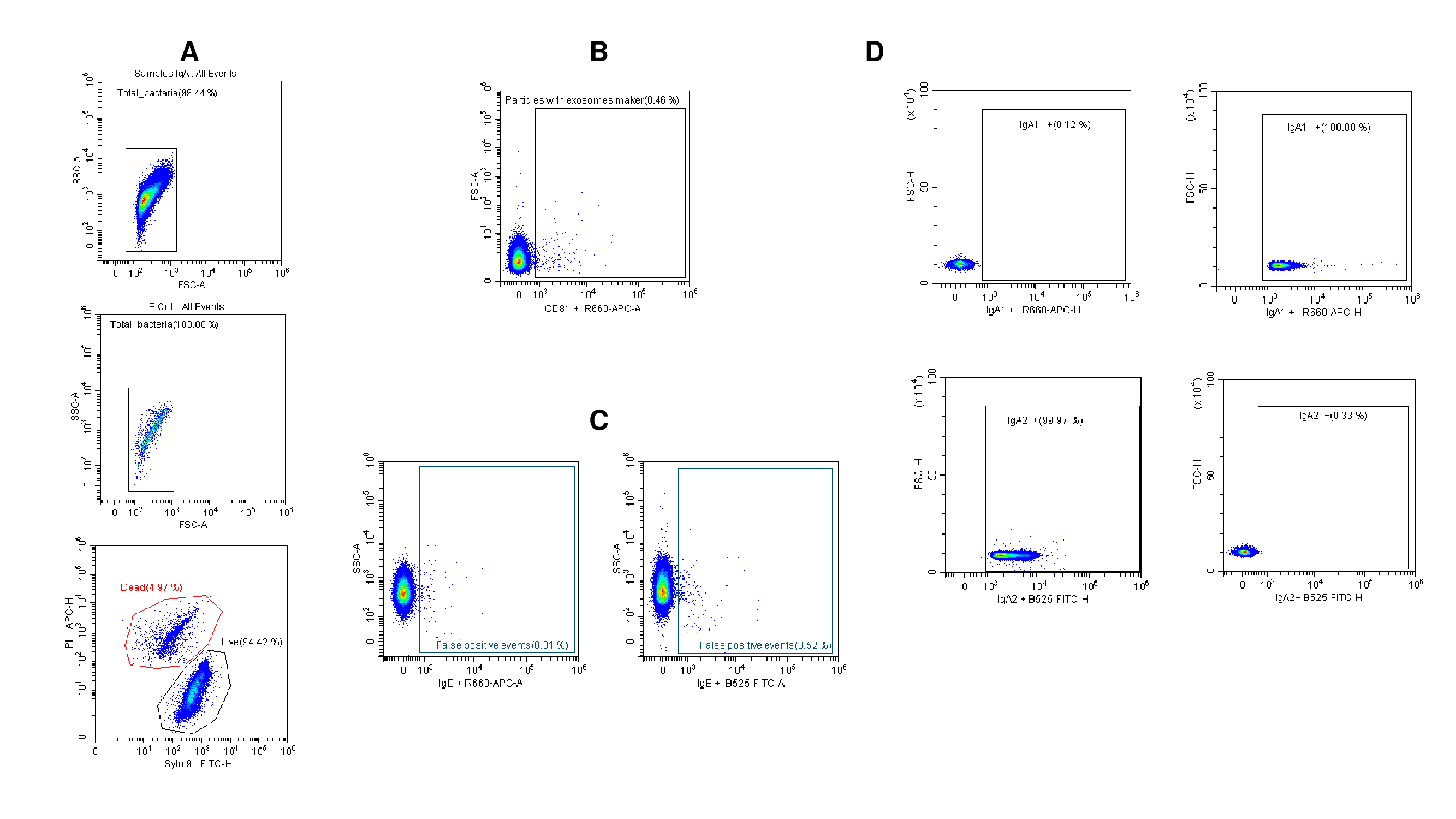

Supplement: Supplementary Figure 1 — Controls for flow cytometry bacterial detection, staining, and magnetic fractionation. (A) Size and complexity profile of total bacteria from samples (top). Dot blot of E coli used as control (middle) and viability assay with Syto9 kit® in bacteria samples (bottom). These parameters were used to adjust gates in size and complexity events into bacterial cells. (B) CD81+ staining control to exclude possible contamination with exosomes. (C) Staining of samples with antihuman IgE as an isotype control, then streptavidin-APC Cy7 (left) and streptavidin-FITC (right). (D) Polystyrene microbeads coated with purified human IgA1 (above) or IgA2 (below); stained with mouse anti-human IgA1 (right) or mouse anti-human IgA2 (left) to show the specificity of the reagents used during bacterial detection, staining, and magnetic fractionation. Figures include the percentage of positive events for each condition. Plots are representative of thirty-six biological samples analyzed. [file Image_1.jpeg]

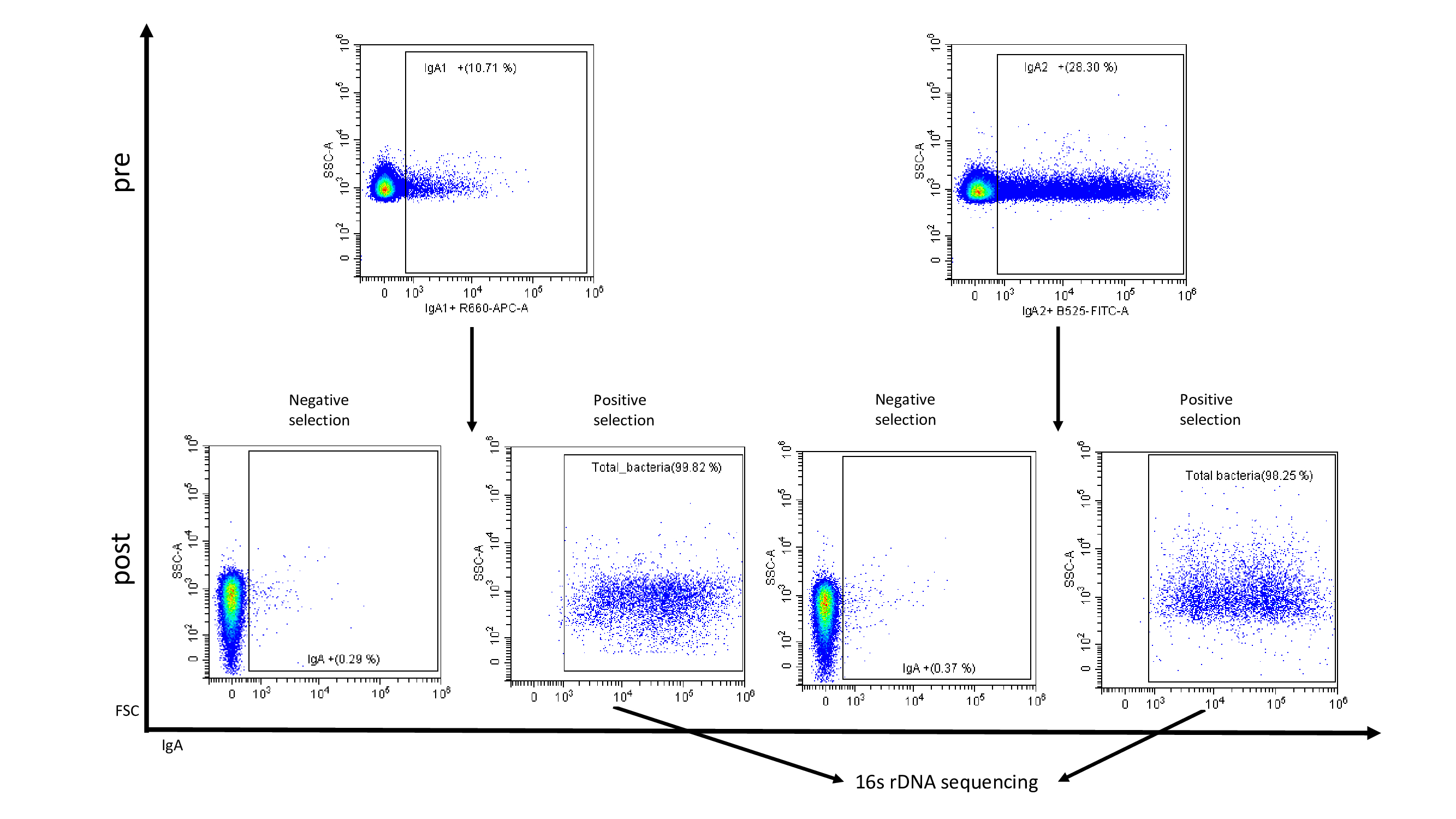

Supplement: Supplementary Figure 2 — Analysis of bacterial populations in colostrum by their associations with IgA1 (APC Cy7) and IgA2 (FITC) in human colostrum, before (above) and after negative (left below) and positive (right below) selections with magnetic beads separation. Plots included the percentage of purity for each condition. Positive selection products were treated for DNA extraction, library preparation, and the massive sequencing process. Plots are representative of thirty-six biological samples analyzed. [file Image_2.jpeg]

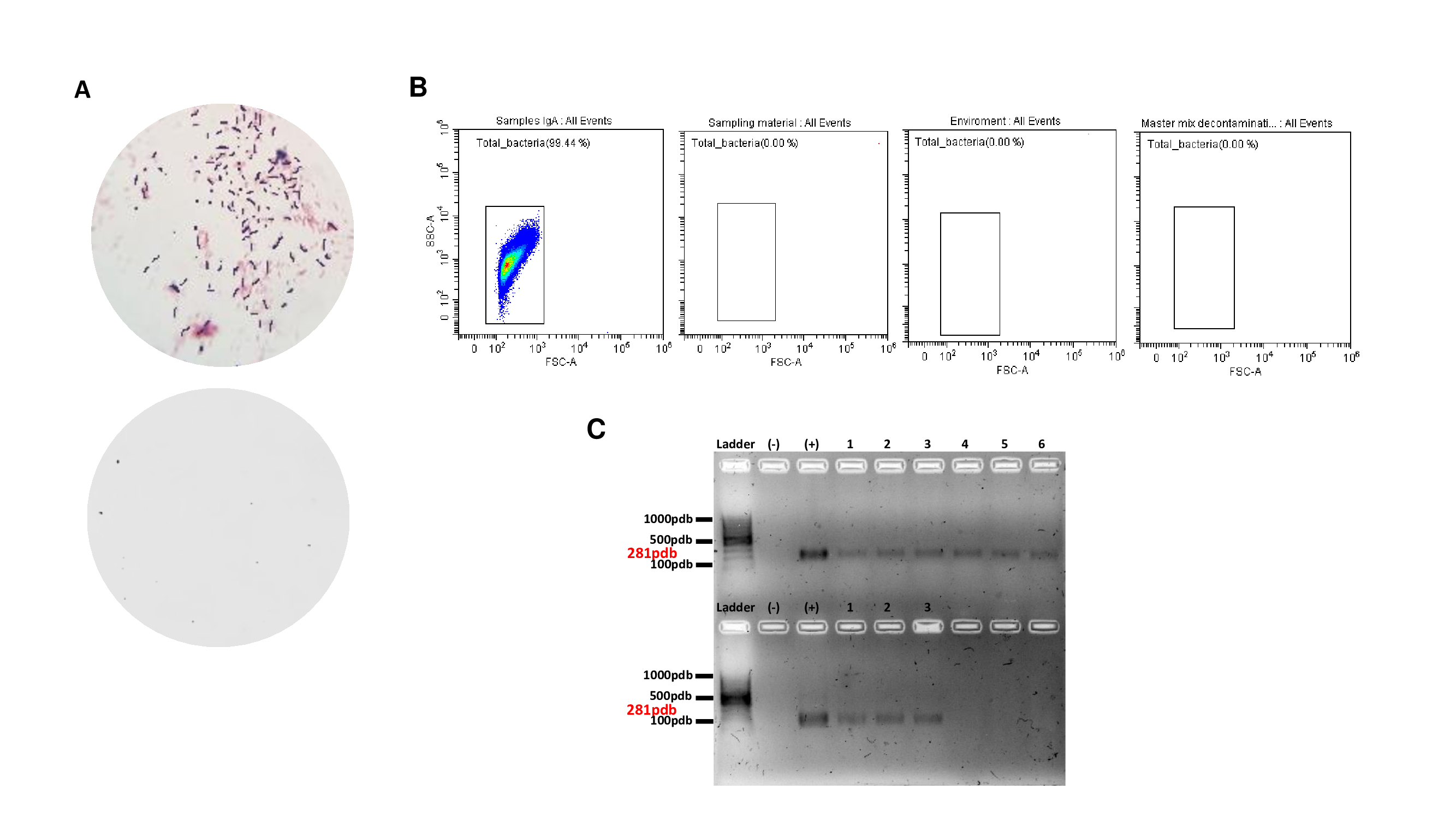

Supplement: Supplementary Figure 3 — Controls for low biomass samples. (A) Gram staining from ten meconium pool samples (above) in comparison with sampling control (below). Slides were prepared with 500 uL of resuspended samples in PBS and dry for 5 minutes before the Gram staining protocol. Magnification = 1000 x. (B) Meconium bacteria sample analyzed by flow cytometry (left) compared with no signal were detected in controls, including sampling material, environment, and Master mix decontamination graphs. (C) The amplification of ~281 bp amplicon was evaluated by electrophoretic fractionation in 2% agarose gel, comparing different six (up) and three (down) meconium samples (numbered as 1-6 and 1-3) with negative (-) and positive (+) controls. These controls consisted of decontaminated PCR mix with sterile water (negative control) and decontaminated PCR mix with DNA extracted from the E coli strain of reference. Controls were included in the mixed pool DNA sequences library for massive sequencing. Ion torrent did not report genetic material detected for sequencing with negative controls. [file Image_3.jpeg]

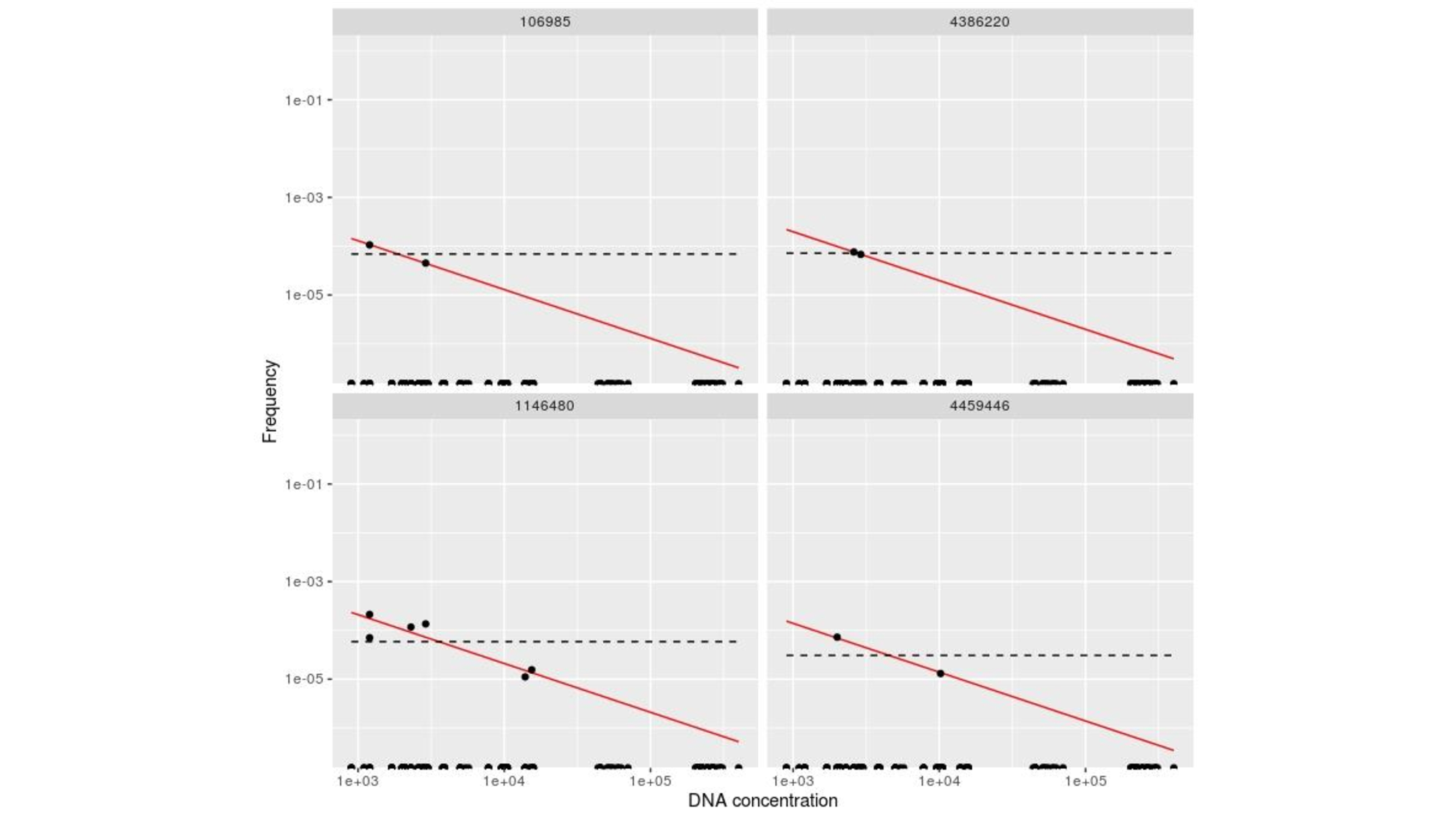

Supplement: Supplementary Figure 4 — Frequency patterns and decontam scores of microbial sequences from 16S rRNA gene dataset. The figure shows frequency patterns of the four most representative OTUs from the twenty-nine “TRUE” results obtained after decontam analysis. These OTUs had the highest frequency values in comparison with the other bacteria from the metadata table. Graphs compare the correlation between DNA concentrations present in the mix library versus frequency values from each sample. Each black dot represents a single sample. [file Image_4.jpeg]

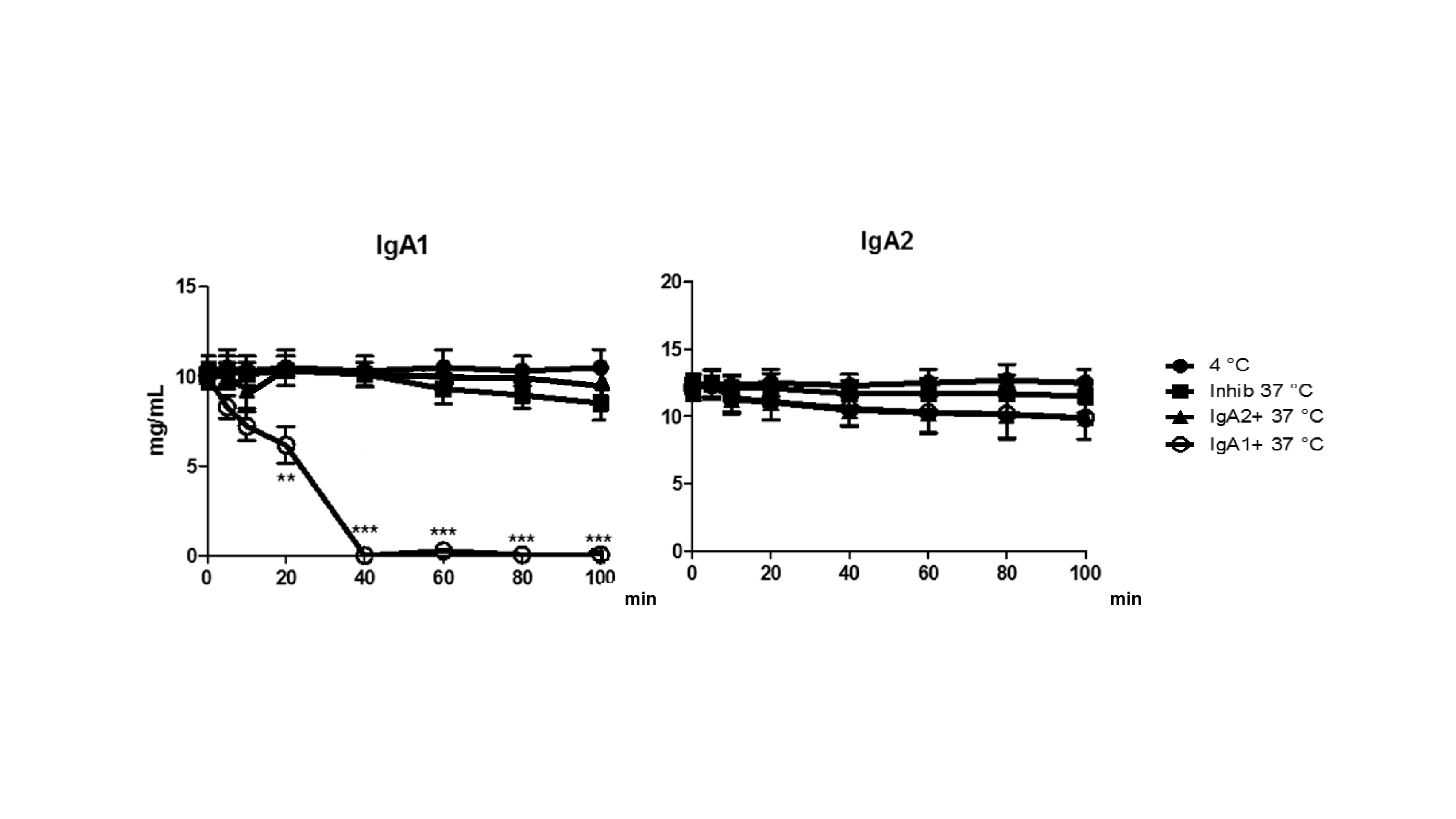

Supplement: Supplementary Figure 5 — Bacterial proteases preferentially degrade IgA1. Kinetical evaluation of the stability of IgA1 (left) and IgA2 (right). IgA subclasses were incubated with IgA2+ or IgA1+ bacteria fractions from colostrum under two different temperature conditions and in the presence or absence of protease inhibitors. The amount of remaining IgA was quantified by ELISA, and the time is expressed in minutes. Each point indicates the mean ± SD of technical triplicates from eight randomly selected samples. All data are expressed in milligrams of IgA subclasses per milliliter (mg/mL). Statistical analysis was performed using the Mann-Whitney U test, comparing each condition at any given time point **p < 0.01 and ***p < 0.001. [file Image_5.jpeg]

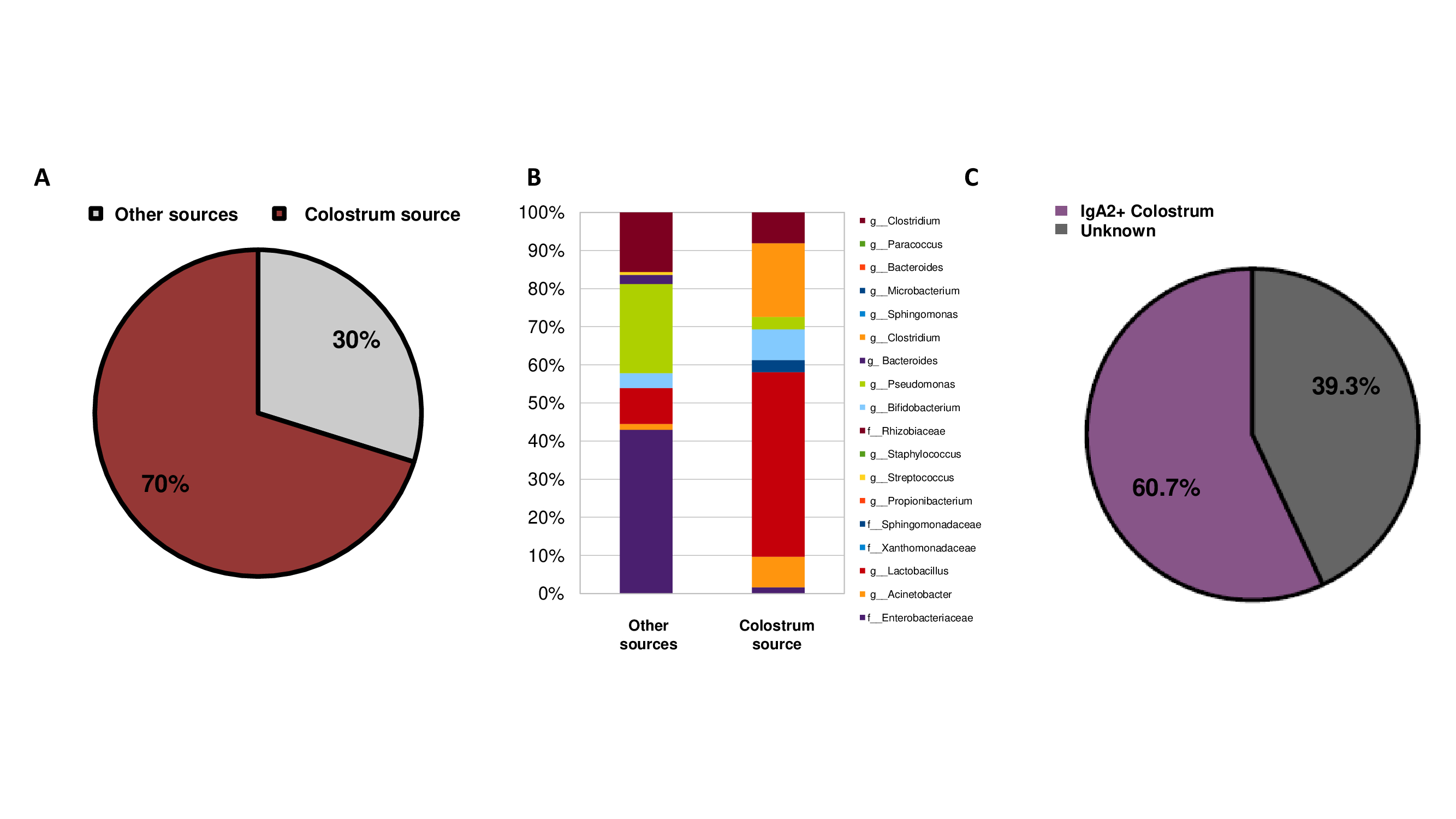

Supplement: Supplementary Figure 6 — Some IgA2+ fecal microbiota founded in the newborn has its origin in the same bacteria fraction from colostrum. (A) Microbial source tracker analysis showing the proportion of bacteria identified in the neonatal stool classified by source. (B) The relative abundance of most common bacterial orders found in the neonatal stool is classified by QIIME source tracker analysis as “Other sources” and “Colostrum source.” Data are from thirty-six samples. (C) Microbial source tracker analysis showing the 60.7% of IgA2+ bacteria identified in the neonatal stool is classified with IgA2+ colostral origin as “IgA2+ Colostrum”. p < 0.001, Wilcoxon signed-rank test. [file Image_6.jpeg]

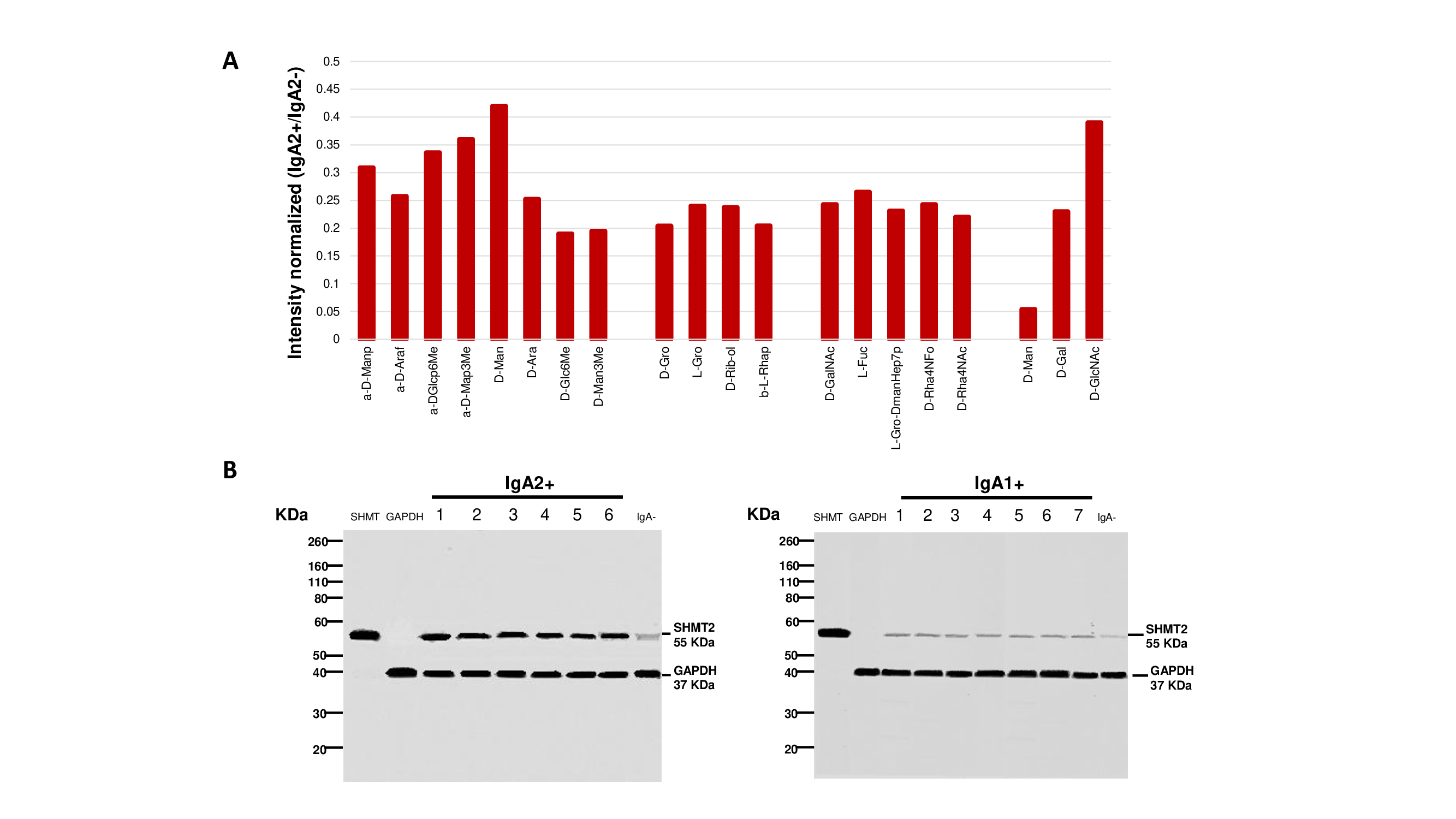

Supplement: Supplementary Figure 7 — IgA2 recognizes SHMT and some glycans on the surface of bacterial microbiota. (A) Bacterial carbohydrates arrangements are recognized by IgA2 from human colostrum. Results are express in normalized OD, comparing purified IgA2 signal from bacteria IgA2+ fraction versus IgA2 signal not associated with bacteria (soluble IgA2). The graph was made with data of triplicates from eight randomly selected samples. (B) Recognition analysis of IgA subclasses in colostrum to SHMT enzyme. Reactivities of six IgA2+ bacteria extracts (left) and seven IgA1+ bacteria extracts, randomly selected (right) from human colostrum, itemized with consecutive numbers. GAPDH was used as a control to confirm that comparable amounts of protein were load in each sample. Images are representative of three independent experimental repetitions. [file Image_7.jpeg]
